# Treatment of a Carbolic Acid Poisoning

**Published:** 1902-08

**Authors:** Francis E. Fronczak

**Affiliations:** Buffalo, N. Y., 508 Fillmore Avenue.


### Treatment of a Carbolic Acid Poisoning.

By FRANCIS E. FRONCZAK, A. M., M. D., Buffalo, N. Y.

AS carbolic acid is one of the swiftest of the deadly poisons, promptness in action is necessarily of utmost importance. No doubt the most common and most easily obtainable antidote of carbolic acid known at present writing is probably alcohol, whether in the form of a staple article or as brandy or whiskey. It is more easily in reach than magnesium sulphate or sodium sulphate, the so-called natural chemical antidotes of carbolic acid, which follow it through the blood and the tissues and there neutralise it by changing into insoluble sulphocarbolates. Hence the first thing to do is to give the patient some brandy or whiskey, about four or five ounces at least. If the patient cannot swallow it, pour it into the stomach through the stomach pump which must be introduced into the pharynx. Then remove the contents of the stomach, and wash it out by either of the soluble sulphates—magnesium or sodium, and also give through the rectum, the solution being in the proportion of an ounce of the sulphate to a pint of water. Sodium sulphate may also be given hypodermatically, not however the magnesium, which when injected into the blood is known to be very poisonous.

Atropine sulphate about 1-60 of a grain should be given hypodermatically every half-hour for several doses (about two or three hours) thus stimulating the respiration which is usually in a very bad condition. I also apply artificial heat to the extremities, and a turpentine stupe to the abdomen for the relief of the pain. The patient having "come to" it is best to feed him for several days by the rectum, and when the stomach is able to receive and retain food, it should be given in the form of liquids, as milk, broths and mucilaginous preparations, as the mucilage of slippery elm. If any complications arise they should be treated secundum artem.

508 Fillmore Avenue.

